# Association of Cardiac Autonomic Dysfunction and Cognitive Impairment With Disease Severity in Parkinson’s Disease: A Cross-Sectional Study

**DOI:** 10.7759/cureus.111116

**Published:** 2026-06-18

**Authors:** Usha Dhanaradja, Rajasegaran Rajalakshmi, Rajeswari Aghoram, Pravati Pal, Sharbari Basu, Moushumi P Mukherjee, Harichandrakumar KT

**Affiliations:** 1 Department of Physiology, Jawaharlal Institute of Postgraduate Medical Education and Research (JIPMER), Puducherry, IND; 2 Department of Neurology, Jawaharlal Institute of Postgraduate Medical Education and Research (JIPMER), Puducherry, IND; 3 Department of Biochemistry, Jawaharlal Institute of Postgraduate Medical Education and Research (JIPMER), Puducherry, IND; 4 Department of Psychiatry, Jawaharlal Institute of Postgraduate Medical Education and Research (JIPMER), Puducherry, IND; 5 Department of Biostatistics, Jawaharlal Institute of Postgraduate Medical Education and Research (JIPMER), Puducherry, IND

**Keywords:** autonomic function, cognition, heart rate variability, non-motor features, oxidative stress, p300, parkinson's disease, reaction time, updrs

## Abstract

Background

Parkinson's disease (PD) is a neurodegenerative disorder characterized by the presence of both motor and non-motor features. Although cardiac autonomic imbalance and cognitive derangement are well documented in PD, their relationship with the severity of the disease has not been explored using a multimodal approach.

Objective

This study aimed to assess the correlation between cardiac autonomic function, cognitive parameters (P300 and reaction time), and biochemical markers (homocysteine (HCY) and 8-hydroxy-2′-deoxyguanosine (8-OHdG)) with disease severity in individuals with mild-to-moderate PD.

Methods

Forty individuals aged 35 to 70 years diagnosed with PD were recruited. Cardiac autonomic function was assessed using heart rate variability (HRV) analysis and conventional autonomic function tests. Cognitive function was evaluated by recording P300 event-related potentials and auditory and visual reaction time. Serum HCY and serum 8‑OHdG were measured as biomarkers of cardiovascular risk and oxidative stress, respectively, using ELISA. Disease severity was assessed using the Movement Disorder Society-Unified Parkinson's Disease Rating Scale (MDS-UPDRS) Part III. Correlation between the study parameters was assessed using the Pearson or Spearman’s rank correlation coefficient test. P<0.05 was considered statistically significant.

Results

Significant positive correlations were observed between UPDRS scores and HRV indices of sympathetic activity (LF power, LFnu), sympathovagal balance (LF/HF), P300 latency, reaction times, and biomarker levels. Significant negative correlations were observed between UPDRS scores and parasympathetic HRV indices (HF power, HF nu), total HRV, conventional autonomic function parameters, and P300 amplitude.

Conclusion

Increasing disease severity in PD is associated with cardiac autonomic imbalance, cognitive impairment, and elevated markers of cardiovascular risk and oxidative stress, suggesting a multi-system involvement.

## Introduction

Parkinson‘s disease (PD) is a progressive neurodegenerative disorder that primarily affects dopaminergic neurons in the substantia nigra, resulting in a wide spectrum of motor and non-motor manifestations. In addition to classical motor symptoms such as bradykinesia, tremor, rigidity, micrographia, and festinating gait, non-motor features, including autonomic dysfunction, cognitive impairment, psychological disturbances, sleep disorders, and anosmia, are increasingly recognized as significant contributors to disease burden. Neuropathologically, PD is characterized by progressive accumulation of cytoplasmic alpha-synuclein in the cortical and subcortical regions as the disease advances. This widespread neurodegeneration underlies the multisystem involvement in PD [1].

The Movement Disorder Society-Sponsored Revision of the Unified Parkinson's Disease Rating Scale (MDS-UPDRS) is the most widely used clinical tool to assess disease severity. It is divided into four components: Part I (Non-Motor Experiences of Daily Living), Part II (Motor Experiences of Daily Living), Part III (Motor Examination), and Part IV (Motor Complications), with each item rated from 0 (normal) to 4 (maximum severity) [2]. The total score from each section provides an overall view of the disease status.

The autonomic dysfunction observed in PD patients is attributed to cardiac and extracardiac denervation. This, in turn, results in untoward alterations in cardiovascular function and baroreflex response, and cardiac autonomic imbalance with increased cardiac sympathetic and decreased cardiac parasympathetic activity [3]. Despite its high prevalence, autonomic dysfunction is usually underdiagnosed and underexplored due to the lack of routine objective autonomic evaluation in clinical practice.

Alterations in cognition are yet another important and clinically relevant non-motor feature of PD. The derangement can span from a mild decline in cognition to severe dementia. PD patients often experience problems in attention, memory, execution, and decision-making, all of which can affect their routine activities and quality of life [4].

Apart from these systemic alterations, recent studies on PD patients have reported elevated levels of homocysteine (HCY), a cardiovascular risk marker, and 8-hydroxy-2′-deoxyguanosine (8-OHdG), a marker of disease severity and progression [5].

Although autonomic dysfunction and cognitive impairment have been individually studied in PD, there is limited evidence examining their simultaneous association with disease severity employing a comprehensive assessment approach. Addressing this gap may improve the understanding of the systemic involvement in PD and facilitate early identification of patients at higher risk of disease progression.

Therefore, the present study aimed to evaluate the association between cardiac autonomic function, cognitive parameters (including P300 and reaction time), biochemical markers (such as HCY and 8-OHdG), and disease severity in individuals with mild-to-moderate PD using a multimodal assessment approach.

## Materials and methods

Ethics approval and study registration

This study was approved by the Institutional Ethics Committee of the Jawaharlal Institute of Postgraduate Medical Education and Research (JIPMER), Puducherry (JIP/IEC/2022/030), and was conducted in accordance with the ethical principles of the Declaration of Helsinki. The study details were explained to the participants, and written informed consent was obtained from all participants before enrolment. For participants with limited literacy, study information was communicated in the local language, and informed consent was obtained via a thumbprint in the presence of an impartial witness, as per institutional ethics committee guidelines and ICMJE recommendations.

The objective of this study was to investigate the association between cardiac autonomic function, cognitive performance, and disease severity as assessed by the MDS-UPDRS score in individuals with PD.

Study design and setting

This was a cross-sectional, observational, analytical study conducted in the Autonomic Function Testing (AFT) Laboratory, Department of Physiology, in collaboration with the Department of Neurology at JIPMER, Puducherry.

Inclusion and exclusion criteria

A total of 40 patients of both genders, aged 35-70 years, with clinically diagnosed PD were recruited. All participants were on stable doses of anti-Parkinsonian therapy for at least three months and had a Movement Disorder Society-Unified Parkinson’s Disease Rating Scale (MDS-UPDRS) Part III motor score of less than 59.

Participants were excluded if they had poorly controlled chronic cardiovascular or respiratory comorbidities, clinically significant dementia as determined by the treating neurologist based on clinical evaluation and screening using the Montreal Cognitive Assessment (MoCA), or other unstable medical conditions that could interfere with study participation or cognitive assessment. In addition, participants with active infections (such as tuberculosis or COVID-19), autoimmune diseases, malignancies, or pre-existing endocrine, hepatic, renal, or connective tissue disorders were also excluded.

Sample size estimation, power, and sampling

The sample size for the parent interventional study was calculated based on the primary outcome variable, the low-frequency to high-frequency (LF/HF) ratio, considering a minimum expected difference of 1 between groups with a standard deviation of 1.5, at a 5% level of significance and 80% power [6]. This yielded an initial sample size of 36 participants per group, which was increased to 43 per group after accounting for an anticipated 15% dropout.

The present study represents a baseline cross-sectional analysis of participants enrolled in this parent study. A total of 40 participants who fulfilled the eligibility criteria were included in the current analysis.

For correlation analysis, a sample size of 40 is sufficient to detect moderate effect sizes (r ≈ 0.4-0.5) with a two-sided significance level of 0.05 and an estimated statistical power of approximately 80% [7]. A consecutive sampling method was followed to recruit PD patients from the Neurology outpatient department.

Assessment of anthropometric and basal cardiovascular parameters

Participants were instructed to report to the AFT laboratory between 08:00 and 11:00 AM, preferably on an empty stomach or after a light breakfast. They were asked to avoid caffeinated beverages for 12 hours and strenuous physical activity for 24 hours prior to the recording. Their demographic details were collected and entered into a data sheet. The height and body weight of the study participants were measured as per standardized protocols [8]. Body mass index (BMI) was calculated using Quetelet's formula (weight (kg) / height (m^2^)).

After five minutes of rest in the supine position, the blood pressure (BP) was recorded using an automated BP apparatus (Omron HEM-8712; Omron Healthcare Co., Ltd., Kyoto, Japan). Two BP readings were recorded at an interval of one minute, and the average was considered for analysis. Mean arterial pressure (MAP) and rate-pressure product (RPP), the measure of myocardial workload, were calculated as follows:

MAP=DBP+1/3 ​(SBP−DBP)

where DBP: diastolic blood pressure; SBP: systolic blood pressure

RPP=Heart Rate × Systolic Blood Pressure×10^−2^

Cardiac autonomic function tests

Short-Term Heart Rate Variability (s-HRV) Analysis

Cardiac autonomic function was assessed using s-HRV analysis as per the Task Force guidelines of the European Society of Cardiology and the North American Society of Pacing and Electrophysiology [9], using the BIOPAC MP36 data-acquisition system (BIOPAC Systems Inc., Goleta, California, USA). Participants were asked to rest in the supine position for 15 minutes in a controlled environment (room temperature ~24°C) prior to recording. A lead II ECG was recorded at 1000 samples/sec for 5 minutes. R-R intervals were extracted and analyzed using Kubios HRV software (version 3.5; Kubios Oy, Kuopio, Finland) to derive time-domain and frequency-domain indices of HRV reflecting sympathetic and parasympathetic activity.

Among the time-domain indices, pNN50 (percentage of NN50 count divided by the total number of all NN intervals), RMSSD (root mean square of successive differences between adjacent NN intervals), and SDNN (standard deviation of normal-to-normal intervals) were analyzed. Among the frequency-domain indices, absolute powers (ms²) in the low-frequency (LF; 0.04-0.15 Hz) and high-frequency (HF; 0.15-0.40 Hz) bands, total power, the LF/HF ratio, and the normalized units of LF (LFnu) and HF (HFnu) were analyzed. LFnu and HFnu were calculated according to Task Force recommendations as:

LFnu = LF/(Total Power − VLF) × 100

HFnu = HF/(Total Power − VLF) × 100

where VLF represents the very low frequency component. The LF/HF ratio was used as an index of sympathovagal balance.

Conventional autonomic function tests

Isometric Handgrip Test

The isometric handgrip test was used to assess sympathetic reactivity. Baseline heart rate and BP were recorded. Maximum voluntary contraction (MVC) was determined using a handgrip dynamometer in the dominant hand. Participants were then instructed to maintain 30% of MVC for four minutes. BP and heart rate were recorded at one-minute intervals during the test, immediately before release, and two minutes after release of the handgrip pressure. An increase in diastolic pressure of more than 16 mm Hg compared to baseline was considered normal, while a value of less than 10 mm Hg was considered abnormal.

Heart Rate Response to Deep Breathing

After five minutes of rest in the supine position, baseline ECG was recorded. Participants were instructed to perform six cycles of controlled deep breathing (inhalation for five seconds followed by exhalation for five seconds). The E: I ratio was calculated as the mean of the longest R-R intervals during expiration to the mean of the shortest R-R intervals during inspiration across six cycles.

Delta HR was calculated as the difference between the maximum HR during inspiration and the minimum HR during expiration, averaged over six cycles.

Assessment of cognition

Assessment of P300 Event‑Related Potential

P300 event-related potentials were recorded using the Nihon Kohden Neuropack M1 EP/EMG system, in accordance with the guidelines of the International Federation of Clinical Neurophysiology [10]. Participants were instructed to attend to the auditory stimuli throughout the recording and to mentally count the infrequent target stimuli. At the end of each run, the participant’s reported count was checked against the expected number of target stimuli. If the count did not match, the run was discarded and repeated. Recordings were repeated until two reproducible trials/waveforms were obtained.

Participants were asked to ensure a clean, oil-free scalp before recording, and earwax was ruled out prior to testing. The ground electrode was placed at Fz, the active electrode at Cz, and reference electrodes over both mastoids. Electrode impedance was checked before recording using the system impedance-check function and was maintained within the acceptable range of < 5 kΩ. An auditory oddball paradigm was used, with infrequent target stimuli presented as tone bursts with 20% probability and frequent non-target stimuli presented as clicks with 80% probability. Stimuli were delivered binaurally through headphones at 40 dB with a stimulation rate of 0.5 Hz. The bandpass filter was set at 0.1-50 Hz.

Signals were amplified, averaged, and analyzed using Neuropack software. Artifact rejection was performed using the Neuropack system’s automatic artifact rejection function, with voltage rejection set according to standard ERP recording practice [10]. Recordings were repeated until two reproducible waveforms averaged across at least 36 artifact-free target responses were obtained. Runs were accepted only when the participant correctly identified the target count. P300 latency, measured in milliseconds, and N2-P3 amplitude, measured in microvolts, obtained from the best trial, were used for analysis.

Assessment of auditory and visual reaction time

Reaction time, an indicator of sensorimotor processing and alertness, was assessed for both auditory and visual stimuli using the RTM-608 Response Analyzer (Medicaid Systems, Chandigarh, India), which has a temporal resolution of 0.1 milliseconds.

Auditory reaction time was recorded using click and tone auditory stimuli generated by the response analyzer. Visual reaction time was assessed using two visual cues, namely red and green light stimuli.

Participants were seated comfortably and familiarized with the testing procedure through practice trials prior to the actual recording. They were instructed to respond as quickly as possible by pressing the response key with the index finger of their dominant hand immediately after perceiving the stimulus. The reaction time values were automatically displayed by the instrument in milliseconds. For each stimulus modality, three recordings were obtained, and the average value was considered for analysis. To minimize anticipatory responses, the interval between consecutive stimuli was randomly varied between two and five seconds.

Assessment of biomarkers

A 5mL sample of venous blood was collected under aseptic precautions. Serum was separated and stored at -20° C until analysis. 

Serum homocysteine (s-HCY) levels were estimated using a commercially available Human Homocysteine (HCy) Competitive ELISA kit (Catalog No. ELK8987) manufactured by ELK Biotechnology Co., Ltd, Sugar Land, USA. The assay was performed according to the manufacturer’s instructions. Optical density was measured at 450 nm using a microplate reader. Quantification was performed using a four-parameter logistic standard curve with standards ranging from 0 to 8000 ng/mL. The assay demonstrated excellent linearity (R² = 0.997). The manufacturer-reported intra-assay and inter-assay coefficients of variation were <10%.

Serum 8-hydroxy-2′-deoxyguanosine (s-8-OHdG) levels were estimated using a commercially available Human 8-OHdG Competitive ELISA kit (Catalog No. E-EL-0028) manufactured by Elabscience, Houston, USA. The assay was performed according to the manufacturer’s instructions. Optical density was measured at 450 nm using a microplate reader. Quantification was performed using a standard calibration curve with standards ranging from 1.56 to 100 ng/mL. The manufacturer-reported intra-assay and inter-assay coefficients of variation were <10%.

Statistical analysis

Data were entered in Microsoft Excel (Microsoft Corporation, Redmond, USA) and analyzed using IBM SPSS Statistics for Windows, Version 22 (Released 2013; IBM Corp., Armonk, New York, United States). Continuous variables are expressed as mean ± standard deviation (SD) or median with interquartile range (IQR), depending on the distribution of the data. Categorical variables are expressed as frequencies and percentages.

The normality of data distribution was assessed using the Kolmogorov-Smirnov test. Variables with normal distribution are presented as mean ± standard deviation (SD), whereas variables with non-normal distribution are presented as median with interquartile range (IQR). Correlation between disease severity (MDS-UPDRS score) and study parameters, namely, cardiac autonomic function indices, cognitive measures, and biochemical markers, was assessed using Pearson or Spearman’s rank correlation coefficient test, depending on the distribution of the data. A P-value <0.05 was considered statistically significant.

## Results

Participant characteristics

A total of 40 participants, 28 male participants and 12 female participants with mild-to-moderate PD, were included in the study. The mean age of the participants was 57.1 ± 8.7 years, with a mean disease duration of 4.0 ± 2.3 years. The mean MDS-UPDRS score was 41.1 ± 5.2, indicating moderate disease severity (Table 1).

**Table 1:** Anthropometric and clinical characteristics of Parkinson’s disease patients (N=40) Values are expressed as mean ± standard deviation. BMI: Body mass index; UPDRS: Unified Parkinson's Disease Rating Scale.

Parameters	Mean (SD)
Age (Years)	57.1 ± 8.7
Height (cm)	159.3 ± 6.0
Weight (kg)	64.7 ± 6.7
BMI (kg/m^2^)	25.5 ± 2.1
UPDRS Score (Disease Severity)	41.1 ± 5.2
Disease Duration (Years)	4.0 ± 2.3

Cardiovascular and autonomic parameters

The baseline cardiovascular parameters and HRV indices are summarized in Table 2. The HRV parameters reveal the distribution of both time-domain and frequency-domain indices indicative of cardiac sympathetic and parasympathetic activity among the study participants.

**Table 2:** Cardiovascular, heart rate variability, and conventional autonomic function test parameters of Parkinson’s disease patients (N=40) ^#^ Values are Median (IQR). SBP: Systolic blood pressure, DBP: Diastolic blood pressure; MAP: Mean arterial pressure; RPP: Rate pressure product; HR (bpm): Heart rate (beats per minute); Mean R-R: Mean duration of R-R interval; pNN50 (%): Percentage of NN50 count divided by the total number of all NN intervals; RMSSD: Square root of the mean of the sum of the squares of differences between adjacent NN intervals; SDNN: Standard deviation of normal-to-normal intervals; LF power: Low-frequency power; LF nu: Low-frequency normalized units; HF power: High-frequency power; HF nu: High-frequency normalized units; LF/HF: Low-frequency-to-high-frequency ratio; TP: Total power. dDBP: difference in diastolic blood pressure; E:I: expiration: inspiration.

Parameters	Mean (SD)
SBP (mmHg)	121.6 (12.7)
DBP (mmHg)	80.9 (10.9)
MAP (mmHg)	94.5 (10.3)
RPP (mmHg/min)	91.4 (14.8)
HR (beats/min)	74.6 (8.5)
Short-Term HRV Indices of Parkinson’s Disease Patients	
Time-Domain Indices of HRV	
RR (ms)	806.8 (108.5)
pNN50 (%)^#^	5.86 (0.23, 18.2)
RMSSD (ms)^#^	27.80 (13.73, 40.8)
SDNN (ms)^#^	25.50 (14.23, 40.73)
Frequency Domain Indices HRV	
LF power (ms^2^)	422.50 (33.56)
LF nu	77.04 (8.76)
HF power (ms^2^)	129.05 (61.36)
HF nu	22.96 (8.76)
LF/HF	3.80 (1.28)
Total power (ms^2^)	577.33 (47.52)
Conventional Autonomic Function Parameters of Parkinson’s Disease Patients	
Isometric Handgrip	
dDBP	15.20 (7.08)
Heart Rate Response to Deep Breathing	
E: I Ratio	1.26 (0.32)

Cognitive parameters

Table 3 summarizes the distribution of cognitive parameters, including P300 latency, P300 amplitude, and auditory and visual reaction times, reflecting electrophysiological and information-processing measures within the study population.

**Table 3:** Cognition parameters of Parkinson’s disease patients (N=40) Values are expressed as Mean (SD).

Parameters	Mean (SD)
Event-Related Potential	
P300 Latency (ms)	317.20 (21.14)
P300 Amplitude (µV)	1.39 (0.26)
Visual Reaction Time	
Red Light (ms)	0.46 (0.07)
Green Light (ms)	0.41 (0.05)
Auditory Reaction Time	
Click (ms)	0.28 (0.05)
Tone (ms)	0.31 (0.05)

Biochemical parameters

Biochemical parameters, including s-HCY and s-8OHdG, are summarized in Table 4, representing the distribution of cardiovascular and oxidative stress-related markers in the study population.

**Table 4:** Biochemical parameters of Parkinson’s disease patients (N=40) Values are expressed as Median (IQR). s-HCY: Serum homocysteine; s-8-OHdG: serum 8-hydroxy-2′-deoxyguanosine

Parameters	Median (IQR)
s-HCY (ng/mL)	821.16 (512.40, 1054.62)
s-8OHdG (ng/mL)	129.59 (27.98, 150.62)

Correlation analysis

Correlation analysis between disease severity (MDS-UPDRS score) and study parameters is presented in Table 5.

**Table 5:** Correlation between UPDRS and cardiac autonomic, cognition and biochemical parameters of Parkinson’s disease patients (N=40) Values are Pearson correlation coefficient. ^#^ Values are of Spearman's rank correlation coefficient. P<0.05 was considered statistically significant. pNN50 (%): Percentage of NN50 count divided by the total number of all NN intervals; RMSSD: Square root of the mean of the sum of the squares of differences between adjacent NN intervals; SDNN: Standard deviation of normal-to-normal intervals; LF power: Low-frequency power; LF nu: Low-frequency normalized units, HF power: High-frequency power; HF nu: High-frequency normalized units; LF/HF: Low-frequency to high-frequency ratio; TP: Total power; dDBP: Difference in diastolic blood pressure; E:I: expiration: Inspiration ratio; s-HCY: Serum homocysteine; s-8OHdG: Serum 8-hydroxy-2’-deoxyguanosine

Parameters	Coefficient		P-value
Correlation between UPDRS and Time Domain Indices of HRV			
Time Domain Indices			
pNN50 (%)^#^	-0.433		0.005
RMSSD (ms)^#^	-0.303		0.057
SDNN (ms)^#^	-0.213		0.186
Correlation between UPDRS and Frequency Domain Indices of HRV			
LF Power (ms^2^)	0.934		<0.001
LF nu	0.867		<0.001
HF Power (ms^2^)	-0.810		<0.001
HF nu	-0.867		<0.001
LF/HF	0.965		<0.001
Total Power (ms^2^)	-0.799		<0.001
Correlation between UPDRS and Conventional Autonomic Function Tests (IHG and DBT)			
Isometric Handgrip			
dDBP (mmHg)	-0.495	0.001	
Heart Rate Response to Deep Breathing			
E: I Ratio	-0.638	<0.001	
Correlation between UPDRS and Cognition Parameters			
Event-Related Potential			
P300 Latency (ms)	0.453		0.003
P300 Amplitude (µV)	-0.953		<0.001
Visual Reaction Time			
Red Light (ms)	0.857		<0.001
Green Light (ms)	0.420		0.007
Auditory Reaction Time			
Click (ms)	0.684		<0.001
Tone (ms)	0.964		<0.001
Correlation between UPDRS and Biochemical Parameters			
s-HCY (ng/mL)^#^	0.862		<0.001
s-8OHdG (ng/mL)^#^	0.807		<0.001

Statistically significant negative correlations were observed between disease severity and parasympathetic HRV indices, including pNN50, HF power, HF (nu), and total power. Similarly, conventional autonomic function parameters, such as the diastolic blood pressure response to isometric handgrip and E:I ratio, also showed significant negative correlations.

Conversely, statistically significant positive correlations were observed between disease severity and indices of sympathetic activity, including LF power, LF (nu), and LF/HF ratio.

Cognitive parameters demonstrated significant positive correlations between disease severity and P300 latency and reaction times, whereas P300 amplitude showed a statistically significant negative correlation.

Biochemical markers, including s-HCY and s-8-OHdG, also showed significant positive correlations with disease severity.

## Discussion

India is home to nearly 0.58 million individuals with PD [11], and this number is expected to increase further in the coming years. PD remains undiagnosed, particularly in early stages, and contributes to disability due to both motor and non-motor manifestations, including autonomic dysfunction, cognitive impairment, bodily dysfunction, psychosocial disturbances, and poor quality of life.

This study was designed to evaluate the association between disease severity (MDS-UPDRS Part III score) and cardiac autonomic function, cognitive performance, and biochemical markers in 40 individuals with mild-to-moderate PD. By adopting a multifaceted approach, this study provides an inclusive overview of the relationship between these parameters in PD.

Studies in the past have reported alterations in the autonomic nervous system in PD, involving both the sympathetic and parasympathetic divisions [3,12,13]. In a study by Bidikar et al., the deep breathing test indicated parasympathetic dysfunction in individuals with PD [14].

In the present study, significant associations were observed between disease severity and several time-domain and frequency-domain indices of HRV and conventional autonomic function tests. HRV parameters reflecting overall heart rate variability (total power) and parasympathetic activity (pNN50, HF nu, and HF power) showed significant negative correlations with disease severity. In contrast, indices reflecting sympathetic activity (LF nu, LF power) and sympathovagal balance (LF/HF) showed significant positive correlations. Additionally, conventional autonomic function parameters, namely, the diastolic blood pressure response to isometric handgrip and the E: I ratio, also demonstrated significant negative correlations with disease severity.

These findings suggest an association between disease severity and alterations in both cardiac sympathetic and parasympathetic nervous system activity. However, these interpretations may be made cautiously due to the absence of a control group. Nevertheless, these findings are in agreement with previous studies that have also reported progressive autonomic involvement with advancing disease [3,12,13].

Cognitive impairment with deficits in attention, executive function, memory, and information processing is an important non-motor feature of PD [4]. It has previously been documented that PD patients exhibit prolonged P300 latency and reduced P300 amplitude, reflecting impaired cognitive processing [15]. In the present study, statistically significant associations were observed between disease severity and cognitive parameters, including P300 latency, P300 amplitude, and both auditory and visual reaction times. These findings suggest that disease severity may be associated with alterations in attention and information-processing domains.

The positive correlation between reaction times and disease severity may indicate motor slowing and delayed cognitive processing. Similarly, alterations in P300 parameters (positive correlation between disease severity and P300 latency and negative correlation between disease severity and P300 amplitude) may reflect alterations in cortical processing and attention. These findings are in agreement with previous reports highlighting progressive cognitive decline in patients with PD [16,17].

The present study evaluated cognitive processing using objective electrophysiological measures, including P300 and reaction time. Although participants were screened for clinically significant cognitive impairment prior to enrollment, future studies combining electrophysiological measures with standardized cognitive assessment tools may provide additional clinical context for characterizing cognitive dysfunction in PD.

It is noteworthy that several HRV-derived parameters demonstrated strong correlations with disease severity. These findings should be interpreted with caution because several HRV indices are derived from the same RR interval series and are physiologically and mathematically interrelated. For example, LFnu and HFnu are normalized measures calculated using a common denominator (total power − VLF), while the LF/HF ratio is directly derived from LF and HF components. Similarly, total power, LF power, and HF power represent overlapping components of HRV, and time-domain measures such as pNN50 and RMSSD reflect related aspects of parasympathetic modulation. Consequently, some strong correlations may partly reflect shared physiological information and mathematical interdependence among HRV variables, in addition to disease-related autonomic alterations. Therefore, these associations should be interpreted as indicators of related autonomic processes rather than independent evidence of distinct biological mechanisms or causal relationships.

Biomarkers of cardiovascular risk and oxidative stress are known to be elevated in PD [5]. While elevated HCY levels have been associated with endothelial dysfunction and neurodegeneration, increased levels of 8-OHdG point towards oxidative DNA damage [18,19].

In this study, significant positive correlations were observed between disease severity and serum levels of HCY and 8-OHdG. These findings suggest an association of disease severity with oxidative stress and cardiovascular risk in PD patients.

Overall, the results of this study highlight the association of autonomic, cognitive, and biochemical alterations with disease severity in PD. The observed correlations suggest that these domains may reflect different aspects of the underlying disease process. In addition, employing a multimodal assessment method in this study provides a more comprehensive understanding of non-motor involvement in PD. It emphasizes the importance of incorporating autonomic and cognitive evaluation into routine clinical evaluation.

Clinical implications

The findings of this study highlight the importance of evaluating non-motor symptoms, including cardiac autonomic function and cognitive performance, in PD patients. In fact, these measures may provide additional insights into overall disease burden.

Incorporation of simple, objective assessments, such as HRV and reaction time measures, may help provide additional insight into autonomic and cognitive alterations associated with disease severity in PD. Furthermore, a multimodal assessment approach may support a more holistic management strategy in PD.

Strengths

A key strength of this study is the adoption of a multimodal, integrated assessment approach to cardiac autonomic function and cognitive status. This has provided an inclusive understanding of non-motor involvement in PD. In addition, the use of objective techniques such as HRV and P300 event-related potentials enhances the robustness and reliability of the findings.

Limitations

This study has several limitations. First, a causal relationship cannot be established between the studied parameters due to the cross-sectional study design. Additionally, the absence of a healthy control group limited direct comparison of the studied parameters with normative values. Second, the small sample size may limit the generalizability of the findings. Third, the analysis did not account for potential confounding variables, such as the relatively broad age range of participants (35-70 years), gender, disease duration, medication use, and educational status, which may have influenced the observed associations. In addition, autonomic and cognitive assessments were not stratified according to ON/OFF medication states, and dopaminergic therapy may have influenced the observed findings. Future studies with larger sample sizes should consider age-adjusted analyses and adjustment for educational attainment and medication-related factors to further validate these findings.

## Conclusions

In conclusion, this study demonstrates significant associations between disease severity and cardiac autonomic function, cognitive performance, and biochemical markers of cardiovascular risk and oxidative stress in individuals with mild-to-moderate PD. These findings highlight the involvement of multiple systems with substantial non-motor features in PD, contributing to overall disease burden.

The study findings also emphasize the importance of incorporating multimodal assessments, including HRV, P300 event-related potentials, and biochemical markers during the clinical evaluation of patients with PD. Such an approach may enhance the understanding of disease severity and associated clinical features. It will also provide additional insights beyond conventional clinical assessment. However, further longitudinal studies with a larger sample size are required to better delineate these relationships and explore their potential clinical applicability.
